# Impact of Different Creatinine Measurement Methods on Liver Transplant Allocation

**DOI:** 10.1371/journal.pone.0090015

**Published:** 2014-02-27

**Authors:** Thorsten Kaiser, Benedict Kinny-Köster, Michael Bartels, Tanja Parthaune, Michael Schmidt, Joachim Thiery

**Affiliations:** 1 Institute of Laboratory Medicine, Clinical Chemistry and Molecular Diagnostics, University Hospital Leipzig, Leipzig, Germany; 2 Department of Visceral, Vascular, Thoracic and Transplant Surgery, University Hospital of Leipzig, Leipzig, Germany; 3 Reference Institute for Bioanalytics, Bonn, Germany; University of Navarra School of Medicine and Center for Applied Medical Research (CIMA), Spain

## Abstract

**Introduction:**

The model for end-stage liver disease (MELD) score is used in many countries to prioritize organ allocation for the majority of patients who require orthotopic liver transplantation. This score is calculated based on the following laboratory parameters: creatinine, bilirubin and the international normalized ratio (INR). Consequently, high measurement accuracy is essential for equitable and fair organ allocation. For serum creatinine measurements, the Jaffé method and enzymatic detection are well-established routine diagnostic tests.

**Methods:**

A total of 1,013 samples from 445 patients on the waiting list or in evaluation for liver transplantation were measured using both creatinine methods from November 2012 to September 2013 at the university hospital Leipzig, Germany. The measurements were performed in parallel according to the manufacturer’s instructions after the samples arrived at the institute of laboratory medicine. Patients who had required renal replacement therapy twice in the previous week were excluded from analyses.

**Results:**

Despite the good correlation between the results of both creatinine quantification methods, relevant differences were observed, which led to different MELD scores. The Jaffé measurement led to greater MELD score in 163/1,013 (16.1%) samples with differences of up to 4 points in one patient, whereas differences of up to 2 points were identified in 15/1,013 (1.5%) samples using the enzymatic assay. Overall, 50/152 (32.9%) patients with MELD scores >20 had higher scores when the Jaffé method was used.

**Discussion:**

Using the Jaffé method to measure creatinine levels in samples from patients who require liver transplantation may lead to a systematic preference in organ allocation. In this study, the differences were particularly pronounced in samples with MELD scores >20, which has clinical relevance in the context of urgency of transplantation. These data suggest that official recommendations are needed to determine which laboratory diagnostic methods should be used when calculating MELD scores.

## Introduction

Because of the severely limited supply of donor livers in Germany, organ allocation is immensely important in the context of liver transplantation.

Prioritization for liver transplantation is based on the model for end-stage liver disease (MELD) score [1], [2], [3].

In Germany, the MELD score has replaced the previously practiced clinically-based organ allocation in 2006 [1] which was based on the Child-Turcotte-Pugh-Score. There is evidence that a reduction in waiting list mortality was achieved by introducing the MELD score [4], [5].

The MELD score is composed of three medical laboratory parameters: bilirubin, creatinine and the international normalized ratio (INR). These parameters are calculated to an integer [1].

The aim of this study was to evaluate the influence of creatinine detection methods on MELD scores and on organ allocation in a cohort of patients who were waiting for liver transplantation. Two well-established detection methods for serum creatinine are available. The Jaffé method is a color reaction in which creatinine and picric acid ion form a red-orange complex that is measured photometrically (Creatinine-J). Alternatively, creatinine concentrations can be measured enzymatically (Creatinine-E) [6].

Depending on the selected method, different interfering variables are possible [7], [8]. In this study, we evaluated the influence of the Jaffé and the enzymatic method on the MELD scores of patients with chronic liver disease that were evaluated for liver transplantation.

## Materials and Methods

A total of 1,109 samples from 463 patients were collected from November 2012 to September 2013. A total of 1,075 samples from 455 patients were available for creatinine measurements using both methods in parallel on the same day of blood taking without any freezing or storing steps before analysis. Creatinine and bilirubin determinations were performed using Cobas 6000 and 8000 analyzers (Roche, Mannheim, Germany) according to the manufacturer’s instructions. Creatinine was measured using the enzymatic assay creatinine Plus Ver. 2 and the creatinine Jaffé Gen. 2 (Roche, Mannheim, Germany).

Bilirubin was measured using the Bilirubin Total DPD Gen.2 kit (Roche, Mannheim, Germany). The prothrombin assay was performed to determine the INR using an ACL TOP 700 System (Instrumentation Laboratory, Lexington, USA) with the RecombiPlasTin 2G kit (Instrumentation Laboratory, Lexington, USA).

All samples were taken from patients on waiting list or from candidates for liver transplantation at University Hospital Leipzig, Germany.

Measurements and MELD diagnostics were performed for routine diagnostics and for reporting to the transplantation organization according to the clinical situation of the patient and the indication was independent of this study. The additional measurements with an additional creatinine method were done for evaluation of the influences on the MELD-score and were approved by the institutional review board of the University of Leipzig. No additional samples for the study were taken. After anonymization of the patients the data from the laboratory information system was analyzed retrospectively. In an additional consultation with the Head of the Ethics Committee of the University Leipzig, it was approved that in this type of retrospective data analysis an additional patient consent was not required.

The MELD scores were calculated from the serum creatinine (mg/dL), serum bilirubin (mg/dL) and prothrombin time (INR) values according to the applicable specifications using the following formula:
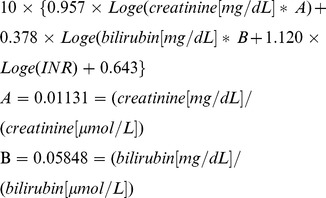



According to the regulations of the Permanent Commission for Organ Transplantation in Germany, creatinine (mg/dL), bilirubin (mg/dL) and INR values that were lower than 1.0 were set to 1.0 for the MELD calculation. The maximum serum creatinine level was set to 4.0 mg/dL = 353.7 µmol/L. Similarly, the maximum creatinine level in dialysis patients was set to 4.0 mg/dL = 353.7 µmol/L. Creatinine values <1.0 mg/dL were set to 1.0 mg/dL = 88.4 µmol/L [9].

### Statistics

Data that were obtained from the bilirubin, creatinine and INR assays were analyzed using Excel 14.0 (Microsoft Corporation, Redmond, USA) and SPSS 20.0 (IBM, Armonk, USA). Pearson’s correlation analysis (bivariate, two-tailed) or a partial correlation analysis (two-tailed) was used to identify any correlations between the parameters. The differences in the mean values were calculated using the *t*-test or the Mann-Whitney U test when appropriate. A *p* value <0.5 was considered statistically significant. The level of agreement between Jaffé und enzymatic creatinine measurements was evaluated according to Bland and Altman [10].

## Results

From November 20, 2012 to September 25, 2013, a total of 1,075 samples from 455 patients were available to determine MELD scores and to measure creatinine enzymatically (Creatinine-E) and according to the Jaffé method (Creatinine-J).

62 samples from 19 patients who had required renal replacement therapy twice in the previous week were excluded from further analyses because according to the applicable rules [9] in these patients creatinine values are fixed at 4.0 mg/dL = 353.7 µmol/L.

The baseline characteristics of the patients are summarized in Table 1.

**Table 1:** The baseline characteristics of the patients and samples in the study.

Number of samples (without RRT)	1,013
Number of patients (without RRT)	445
Patient sex	Men: 254/445 (57.1%)
	Women: 191/445 (42.9%)
Patient age (median, years)	57
*Range (years)*	*22–76*
Median enzymatic creatinine µmol/L (mg/dl)	76 (0.86)
*Range µmol/L (mg/dl)*	*20–434 (0.23–4.91)*
Median Jaffé creatinine µmol/L (mg/dl)	80 (0.90)
*Range µmol/L (mg/dl)*	*28–431 (0.32–4.87)*
Median bilirubin µmol/L (mg/dl)	26 (1.52)
*Range µmol/L (mg/dl)*	*2.2–1096.8 (0.13–64.14)*
Median INR	1.3
*Range*	*0.8–9.0*
Median MELD-E score	11
*Range*	*6–40*
Median MELD-J score	11
*Range*	*6–40*
Number of samples with MELD scores (MELD-E)	
*6–10 points*	442
*11–20 points*	419
*21–30 points*	124
*31–40 points*	28
Number of samples with a maximum MELD score >40 (enzymatic and Jaffé creatinine measurement methods)*	4

*According to the applicable regulations [9] MELD scores were limited to 40 and rounded to an integer before being reported to the transplantation organization. The samples had MELD scores >40 independently from the method that was used for creatinine measurement.

**Figure 1:**
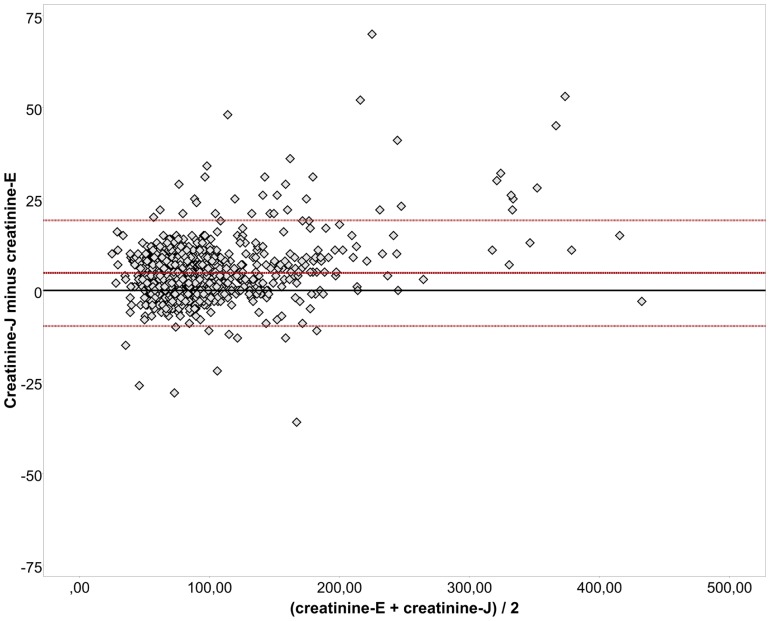
Bland-Altman-plot shows the differences between the creatinine measurements determined using the Jaffé method (creatinine-J) and the enzymatic method (creatinine-E) versus the mean measurements of both methods. The horizontal reference lines show zero difference, the average difference between the measurements and the measurements plus and minus a 1.96-fold standard deviation [10].

Overall, a good correlation was observed between the enzymatic and Jaffé creatinine methods (creatinine-E and -J) (r = 0.989; *p*<0.001). However, remarkable differences were detected in many of the samples from candidates for liver transplantation and from patients on a waiting list. The Bland-Altman plot revealed systematic deviations in the higher levels of serum creatinine (Fig. 1).

**Figure 2:**
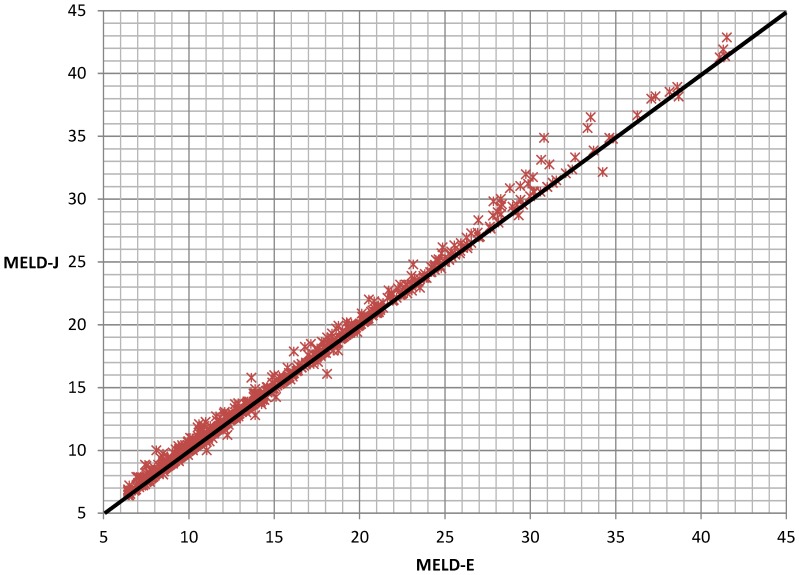
Correlation between MELD-J and MELD-E results. For this figure, we did not restrict the MELD scores to a maximum of 40 points, and values were not rounded (r = 0.998; *p*<0.001).

**Figure 3:**
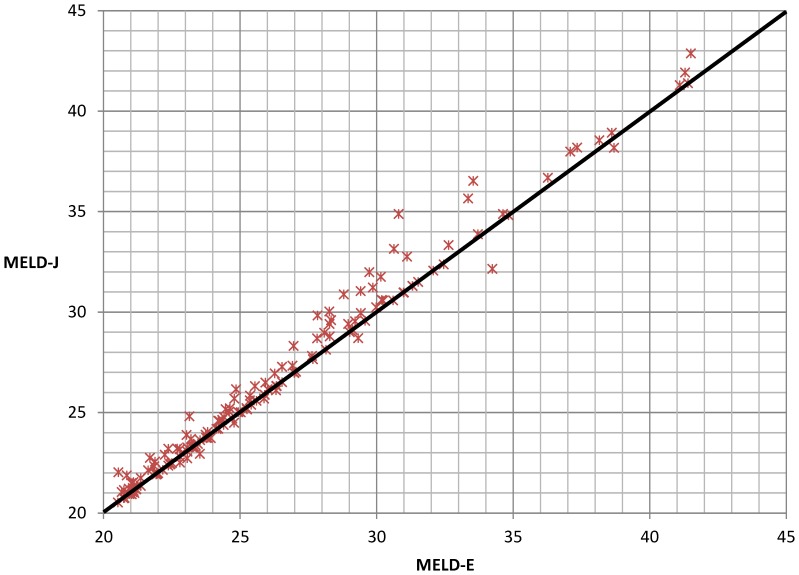
Correlation between MELD-J and MELD-E results for MELD-E scores >20. For this figure, we did not restrict the MELD scores to a maximum of 40 points, and values were not rounded (r = 0.992; *p*<0.001).

Consequently, the MELD scores that were calculated using the results of the enzymatic creatinine assay (MELD-E) correlated with the MELD scores that were calculated using the results of the Jaffé assay (MELD-J) (Fig. 2). This correlation becomes increasingly imprecise for samples with MELD scores >20 (Fig. 3).

According to the applicable regulations [9], MELD scores were limited to 40 and rounded to an integer before being reported to the transplantation organization.

MELD-E and MELD-J scores did not vary for most of the samples (835/1,013 = 82.4%). Jaffé measurements led to greater differences in the MELD scores in 163/1,013 (16.1%) samples with up to a 4-point difference in one case. Enzymatic measurements led to greater differences in the MELD scores in 15/1,013 (1.5%) samples with up to a 2-point difference in two cases (*p*<0.001). Table 2 shows the differences between the MELD scores that were obtained when creatinine was measured enzymatically (MELD-E) and according to the Jaffé method (MELD-J) in detail.

**Table 2:** MELD scores for the creatinine measurements that were obtained using the enzymatic assay (MELD-E) and the Jaffé method (MELD-J).

Difference between MELD-E andMELD-J scores	Number of samples with higherMELD-E scores (%)	Number of samples with higherMELD-J scores (%)
**0**	835/1,013 (82.4%) samples exhibitedno differences	
**1**	13 (1.3%)*	143 (14.1%)*
**2**	2 (0.2%)**	17 (1.7%)**
**3**	0 (0.0%)	2 (0.2%)
**4**	0 (0.0%)	1 (0.1%)
**Total**	15/1,013 (1.5%)**	163/1,013 (16.1%)**

(*p<0.001; **p = 0.001).

Overall, 2 samples had higher MELD-J scores than MELD-E scores; however, because the MELD scores were limited to 40, the MELD results did not differ.

**Figure 4:**
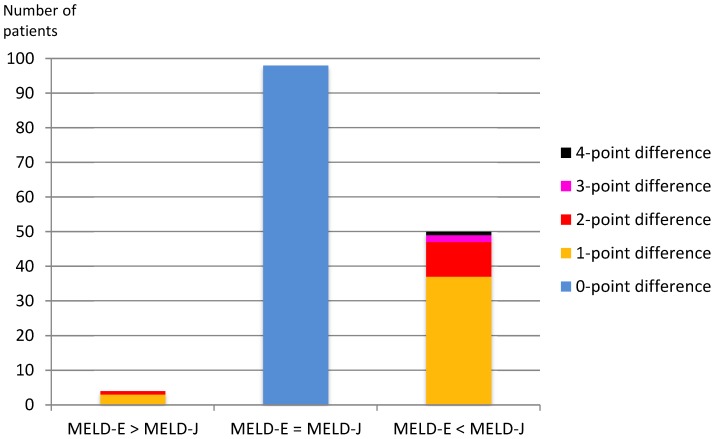
A comparison of the creatinine measurements that were obtained enzymatically (MELD-E) and according to the Jaffé method (MELD-J) for samples with MELD scores >20. Overall, 152/1,013 (15.0%) samples met this condition.

Importantly, in the samples from patients with MELD scores >20, the differences between MELD-E and MELD-J scores were more pronounced (Table 3). For a better illustration Figure 4 shows the high proportion of the samples with higher MELD-J scores in relation to samples with equal MELD scores or higher MELD-E scores.

**Table 3:** A comparison of the creatinine measurements that were obtained enzymatically (MELD-E) and according to the Jaffé method (MELD-J) for samples with MELD scores >20.

Difference between MELD-E andMELD-J scores	Number of samples with higherMELD-E scores (%)	Number of samples with higherMELD-J scores (%)
**0**	98/152 (64.5%) samples exhibited no differences	
**1**	3 (2.0%)	37 (24.3%)
**2**	1 (0.7%)	10 (6.6%)
**3**	0 (0.0%)	2 (1.3%)
**4**	0 (0.0%)	1 (0.7%)
**Total**	**4/152 (2.6%)**	**50/152 (32.9%)**

The highest discrepancy between the MELD results was 4 points in 1 case (Tables 2, 3). The corresponding MELD-E- and MELD-J scores were 31 and 35. Remarkably, all of the samples with differences of more than 2 MELD points had higher creatinine levels when measured using the Jaffé method.

To identify possible confounders for the enzymatic and Jaffé creatinine measurements, we performed a linear correlation analysis of the differences between the MELD-E and MELD-J scores and other routinely measured parameters, including creatinine, bilirubin, INR, MELD-E, albumin, total protein, sodium, osmolality, glucose, and C-reactive protein (CRP) CRP. A total of 1,013 measurements were available for creatinine-E and creatinine-J, bilirubin, INR and MELD-E. A total of 930 measurements were available for sodium, followed by 767 for osmolality, 907 for albumin, 845 for protein, 858 for glucose and 922 for CRP.

We found significant correlations between the MELD differences and creatinine-E (r = 0.219; *p*<0.001), creatinine-J (r = 0.295; *p*<0.001), bilirubin (r = 0.234; *p*<0.001), INR (r = 0.129; *p*<0.001), MELD-E (r = 0.243; *p* = <0.001) and sodium (r = −0.107; *p* = 0.001). No significant correlations were found between MELD differences and osmolality, albumin, total protein, glucose or CRP.

A partial correlation analysis, which was controlled for MELD-E scores, confirmed the results. After controlling for bilirubin and INR, the correlations between MELD differences and INR and between MELD differences and bilirubin were no longer significant (*p*>0.5).

## Discussion

In this study, we demonstrate that creatinine measurement method affected MELD scores, which has an important and direct impact on organ allocation.

In 178 of 1,013 samples (17.6%) from 119 different patients differences between MELD-E and MELD-J scores were detected. Most of the differences between MELD scores were one point. Importantly, these differences were particularly pronounced in the patients with a MELD score >20. Because transplantations are usually performed in patients with a MELD score >20, this finding has direct clinical relevance.

All of the samples with differences >2 points had higher MELD scores when creatinine was measured using the Jaffé assay. The maximum difference was 4 MELD points in one patient. Using this assay in our cohort would lead to a systematic preference for organ allocation.

Influences of different creatinine detection methods on MELD scores have been observed in smaller studies before [11], [12].

But in contrast to previous studies we only included samples of patients that were on the waiting list or in evaluation for liver transplantation. Furthermore creatinine measurements in this study were performed with different methods in parallel on the day of blood taking without any freezing or storing steps before analysis. For this reason we can exclude additional preanalytical influences that would affect the concentration of interfering variables as ketones for example.

An important limitation of this study is that we only used the assay of one manufacturer, and the results of a reference method for an additional determination of creatinine were not available.

For this reason, we can only speculate if methods of other manufacturers would be more suitable for patients for liver allocation. A standardization of diagnostic laboratory MELD scores is urgently needed to prevent a systematic preference or discrimination of liver transplant recipients.

There is already some evidence available that laboratory methods have relevant influences on the MELD score for patients with liver diseases. Importantly variations in the method of INR diagnostics seem to have an even more pronounced influence on the MELD score than the described method of creatinine measurement [13], [14], [15].

Therefore, in cooperation with the Reference Institute for Bioanalytics in Germany (http://www.dgkl-rfb.de), we initiated recently a proficiency test to compare the MELD scores that are obtained in diagnostic laboratories as part of medical laboratory quality assurance, especially for laboratories at transplant centers. These results will provide a basis for further standardization efforts.

The results of this study support the need to standardize the laboratory methods for obtaining MELD scores. In our opinion official recommendations are urgently needed from transplantation organizations regarding the obligatory creatinine measurement method.

Presently the Jaffé method remains the most common creatinine measurement method. According to our inquiries to the manufacturer of the assays that we used in this study, the proportion of laboratories that use the Jaffé method vs. the enzymatic assay in Germany and worldwide is approximately 70% vs. 30%, respectively. To verify this information we analyzed the data from proficiency tests of the Reference Institute for Bioanalytics (RfB) in Germany (http://www.dgkl-rfb.de). In 2013 eight independent proficiency panels for creatinine measurement with a total of 5,038 participants from Germany were performed. 3,951 participants (78.4%) used the Jaffé method and 1.087 (21.6%) used the enzymatic method. A reason for this finding might be that the Jaffé method is more affordable than the enzymatic assay.

The reasons for the discrepancies between creatinine values in patients who require liver transplantation are not entirely known. The manufacturer of the assays that were used in this study lists several limitations and interferences to the creatinine measurement methods in their manuals (Table 4).

**Table 4:** Relevant confounders that limit creatinine measurement accuracy according to manufacturer’s test manuals for the enzymatic (creatinine-E) and Jaffé assay (creatinine-J).

Relevant influences	Creatinine-E measurement	Creatinine-J measurement
Icterus	• Values above 257 µmol/L (15 mg/dl) for conjugated bilirubin and above 342 µmol/L (20 mg/dl) for unconjugated bilirubin [18]	• Values above 86 µmol/L (5 mg/dl) for conjugated bilirubin and above 171 µmol/L (10 mg/dl) for unconjugated bilirubin [18]
Hemolysis	• Values above 497 µmol/L (800 mg/dl) for hemoglobin [18]	• Values above 621 µmol/L (1,000 mg/dl) for hemoglobin [18]
	• HbF values above 600 mg/dl [19]	• HbF values above 60 mg/dl [19]
Lipemia	• L-index* above 2,000 [18]	• L-index* above 800 [18]
Medication:		
• *Incorrect low*	• Rifampicin	
	• Levodopa	
	• Calcium Dobesilate	
• *Incorrect high*	• DL-Proline values above 1 mmol/l (115 mg/l)	• Cefoxitin
	• N-Ethylglycine	
• *Both directions*	• Ascorbic acid values above 1,70 mmol/l (300 mg/dl)	• Cyanokit (hydroxocobalamine) [20], [21]
	• Phenindione [20], [21]	
Ketones	• No influence	• Influence due to pseudo-creatinine chromogens [22]
Others	• Rarely gammopathy (especially type M, Waldenström’s macroglobulinemia) may influence both measurement methods	

*The L-index corresponds to the blur of the probe. There is no exact correlation with the triglyceride concentration.

An important difference between the tests that we used in the study is that concentrations above 86 µmol/L (5 mg/dl) for conjugated bilirubin and above 171 µmol/L (10 mg/dl) for unconjugated bilirubin interfere with the Jaffé method according to the instructions in the manufacturer’s manuals. For the enzymatic measurement, relevant interferences are expected for values above 257 µmol/L (15 mg/dl) for conjugated bilirubin and above 342 µmol/L (20 mg/dl) for unconjugated bilirubin. Another difference is a potential discrepancy in the creatinine-J results due to unspecific reactions with pseudo-creatinine chromogens, such as proteins and ketones. According to the manufacturer’s manual, 26 µmol/L (0.3 mg/dl) was automatically subtracted from the results during the measurement of the Jaffé assay.

However, these confounders do not completely explain the observed differences. Only a few other publications have focused on this topic. Greenberg et al. recently published on the specificity characteristics of 7 commercial creatinine measurement procedures [16]. They found that serum glucose, albumin and total protein levels significantly influenced the creatinine results, especially for the Jaffé method. Lower protein and albumin levels are frequent in patients with advanced liver disease and would lead to higher MELD scores when the Jaffé method is used to assess creatinine levels. This influence was confirmed by Kuster et al. [12].

In our study, we could not confirm the previously described influence of glucose, albumin and total protein on creatinine measurements. Our data suggest that the Jaffé assay that we used in our study may be less influential due to optimizations by the manufacturer [11], [16]. Therefore, the differences between the creatinine results in liver cirrhotic patients may be even more pronounced using other assays.

The underlying causes for the differences between the enzymatic and Jaffé measurements of creatinine remain unclear. The correlation analyses indicated significant correlations between the MELD score differences and MELD-E, creatinine-E, creatinine-J, bilirubin, INR and sodium results. Interestingly, all of these parameters were important prognostic parameters for advanced liver diseases. Creatinine, bilirubin and INR are components of the MELD score, and the addition of sodium as an independent risk factor has resulted in a better prediction of mortality in these patients [17].

A partial correlation analysis, which was controlled for MELD-E scores, confirmed the results. It could be assumed that other components in the serum of patients with liver diseases may play an important role in the measurement accuracy of these methods and the prognosis of patients.

As a consequence of the presented data the liver transplantation guidelines should be concretized regarding the measurement method for creatinine. In our opinion Iaboratory investigations should be performed in a harmonized process with the enzymatic method to reduce derivations between transplantation centers and to improve the fairness of the allocation process.
